# Incidence of differentiated thyroid cancer in Canada by City of residence

**DOI:** 10.1186/s40463-015-0088-0

**Published:** 2015-09-15

**Authors:** Martin J. Corsten, Matthew Hearn, James Ted McDonald, Stephanie Johnson-Obaseki

**Affiliations:** Department of Otolaryngology – Head and Neck Surgery, Aurora St. Luke’s Hospital, Suite 630, 2801W Kinnickkinnik River Parkway, Milwaukee, WI 53215 USA; Department of Otolaryngology-Head and Neck Surgery, University of Ottawa, S3 – 501 Smyth Road, Ottawa, ON K1H 8L6 Canada; Department of Economics, University of New Brunswick, PO box 4400, Fredericton, NB E3B6C4 Canada

**Keywords:** Thyroid cancer, Incidence, Geographic distribution, City, Rural, Urban

## Abstract

**Background:**

Thyroid cancer incidence in Canada is increased in high socioeconomic groups, and in urban compared with rural areas. The objective of this study was to analyze patterns in thyroid cancer incidence across Canada, particularly with respect to the major urban areas across the country, to identify whether there are any discrepancies in thyroid cancer incidence between Canadian cities.

**Methods:**

Cases were drawn from the Canadian Cancer Registry. Demographic and socioeconomic information were extracted from the Canadian Census of Population data. We linked cases to income quintiles (InQs) by patients’ postal codes, and categorized residence by census metropolitan area ((CMA), population >100,000). Within the Toronto CMA we further classified by census subdivision (CSD).

**Results:**

There were a total of 33 CMAs across the country. After controlling for demographic and socio-economic factors, we found that the Toronto CMA had an IRR of thyroid cancer that was significantly higher than all other CMAs across the country. For 70 % of CMAs and CAs across Canada, the IRR for thyroid cancer was less than half of the IRR for thyroid cancer in the Toronto CMA.

As Toronto is one of the largest CMAs, we then subdivided the Toronto area into CSDs to examine how incidence of thyroid cancer varies within this large area. The Toronto City core was used as the reference category and all other areas were compared directly to it. In doing so, we found that a contiguous area of three CSDs North of Toronto had higher IRRs compared with the Toronto city core: Markham, Vaughan and Richmond Hill.

**Conclusions:**

After controlling for demographic and socioeconomic factors, we found that the Toronto CMA has the highest incidence of thyroid cancer nationwide. Several explanations could account for this discrepancy including increased detection due to increased access to imaging, differences in ethnicity or environmental exposures.

## Background

Thyroid cancer is a commonly encountered disease entity. The American Cancer Society estimates the incidence of thyroid cancer in the United States at approximately 62,000 new cases annually [1]. Thyroid cancers are much more commonly found in women than men. The vast majority of these cancers fall into the category of differentiated thyroid cancer (DTC), which includes the papillary and follicular subtypes. DTC represents approximately 97 % of thyroid cancers [2].

Over the last decade a number of studies have demonstrated a dramatic increase in the incidence of thyroid cancer [1–4]. In the United States, the National Cancer Institute’s Surveillance, Epidemiology, and End Results (SEER) database demonstrated a 2.4 fold increase in the incidence of thyroid cancer between 1973 and 2002 [2]. In Canada, a similar trend has been observed. The Canadian Cancer Statistics Report has shown that the annual incidence of thyroid cancer in Canadian women increased at a rate of 7 % per annum from 2002 to 2012 [5]. Multiple factors have been proposed to explain this increase in thyroid cancer incidence, including increased detection with ultrasound, exposure to radiation, changing reproductive patterns in women, increased incidence of obesity, and changes in the pathological classification of thyroid masses [2, 6–13].

Our group previously demonstrated a relationship between thyroid cancer incidence, socio-economic status and population density (urban vs. rural residence). We demonstrated a higher incidence in Canada of thyroid cancer in patients living in neighborhoods with higher average incomes, and in cities as compared to towns or rural areas [14]. These disparities in the incidence of thyroid cancer were supported in a recent study by Hall et al. Their group found that thyroid cancer incidence rates differ between Local Health Integration Networks (LHINs) in Ontario – which are subdivisions within the province in which healthcare is delivered. They also found that there was a positive correlation between incidence of thyroid cancer and rates of diagnostic imaging studies, especially neck ultrasound [15].

The purpose of this study was to further analyze patterns in thyroid cancer incidence across Canada, particularly with respect to the major urban areas across the country, to identify whether there are any discrepancies in thyroid cancer incidence in Canadian cities.

## Methods

Two different sources were used to draw our data: the Canadian Cancer Registry (CCR) and the Canadian Census of Population from Statistics Canada [16]. The CCR data file contains patient demographic and tumor – specific data included in provincial and territorial cancer registries from 1992 to 2007 inclusive. It provides information on all cases of cancer diagnosed in individuals whose usual place of residence is Canada including all rural and urban areas of each province and territory [16]. Thus, observed differences in the incidence of thyroid cancer between urban and rural areas reflect differences in the extent to which cancers are being diagnosed, differences in the actual incidence of the disease or both. Names and personal identification numbers were removed and replaced with unique identifiers in the version of the file released for this study. All analysis was within the University of New Brunswick Research Data Centre (NB-RDC), and all output was vetted for release using enhanced vetting methods required by Statistics Canada. Ethics approval is not required for research projects using data stored in the NB-RDC. To our knowledge, this is the first time CCR data have been available and used to study the relationship between thyroid cancer incidence and SES in Canada.

The Long Form of the Canadian Census of population occurs every five years and contains demographic and socioeconomic data on 20 % of the Canadian population. For this study, data from the census years (CYs) 1991, 1996, 2001 and 2006 were available. The narrowest level of disaggregation at which census information is released by Statistics Canada is the dissemination area (DA), a small, relatively stable geographic unit composed of one or more dissemination blocks, including a population of 400–700 individuals. For each DA and for each of the four CYs, information was collected on age for men and women, average household income, proportion of adult residents with at least a university degree, and other socio-demographic information such as the proportion of the population born outside of Canada or belonging to an ethnic minority group. Although average income in the DA might be influenced by large income outliers, median income is not available at the DA level for all of the CYs. Each DA is assigned to one of three categories of rural/urban status based on definitions provided by Statistics Canada: city, if the DA is located in a census metropolitan area (CMA; an area with a total population of at least 100,000 of which 50,000 or more must reside in an urban core); town, if the DA is located in a census agglomeration (CA; an area with a total population of between 10,000 and 100,000); and rural, if the DA is not located in either a CMA or CA. The number of DAs for which census information was available was 32,825 in 1991, 38,016 in 1996, 46,909 in 2001 and 52,443 in 2006. In order to control for wage and price differences across different regions, the income quintile (InQ) for each DA was defined relative to other DAs in the associated census division (CD), which Statistics Canada defines as a group of neighboring municipalities joined together for the purpose of regional planning and managing common services. The number of CDs in Canada was in the range of 288–290 across the four CYs, and changes occurred to only a few CD boundaries each CY. DAs within each CD were sorted by average income then assigned to one of five InQs. Although the DA of an individual in the CCR database is not reported, the postal code of residence is disclosed. The Postal Code Converter File (PCCF+) is a sophisticated statistical tool provided by Statistics Canada that maps postal codes to DAs. Statistics Canada revises geographic boundaries and updates PCCF+ at each CY. Hence, in order to match postal code and DA as closely as possible, cancer cases were assigned to CYs as follows: cases diagnosed in the years 1992–1995 were paired with socioeconomic characteristics according to the 1991 census; cases diagnosed in the years 1996–2000 were associated with the 1996 census; cases diagnosed in the years 2001–2005 were associated with data of the 2001 census; and cases diagnosed in the years 2006–2007 were associated with data from the 2006 census. Census data could be linked to 96.9 % of DAs for the CY 1991, 97.4 % for the CY 1996, 99.0 % for the CY 2001 and 99.2 % in the CY 2006.

For each CY, the unit of observation for the analyses of incidence was the DA and the key variable was the number of cases of thyroid cancer diagnosed in adults over the age of 18 in each DA over a relevant period of time corresponding to the CY. At this level of geography, it was not feasible to disaggregate counts further by age and sex. In the regression analysis, controls were included for the age and sex composition of the adult population of each DA. The exposure variable was the adult population in the DA at the CY multiplied by the number of years in the corresponding time interval for that census (2, 4, or 5 years). Negative binomial regression models were estimated where neighborhood socioeconomic status, as measured by the InQ of the DA, was captured by a binary variable for each InQ, with the highest InQ specified as the baseline. To capture the changes in incidence over time, we defined indicator variables for each CY from 1996 to 2006 with 1991 as the reference year. In our first specification, differences in rural or urban status were captured by a binary variable for each type of region defined above. In our second specification, we interacted the InQ variables with indicators for residence in cities, towns and rural areas to allow the relationship between socioeconomic status and incidence of thyroid cancer to vary by urban–rural residence. Both regressions included detailed controls for the age and sex composition of the DA. Since healthcare in Canada is administered at the provincial level, both regressions also included indicator variables for each province or territory of residence in order to capture regional unobserved effects important to the incidence and diagnosis of thyroid cancer.

Incidence models included binary indicator variables for each CMA and CA in Canada, with Toronto CMA as the omitted category. Incidence models also included controls for the age and sex distribution of the DA, the InQ of the average household income of the DA as well as the proportion of adults residing in the DA who has a university degree, indicator variables for census year and indicator variables for province.

## Results

After performing our initial binomial regression for the census years of 1996–2006 and categorizing results by city of residence, we note that there were roughly comparable rates of incidence of thyroid cancer amongst the vast majority of the census metropolitan areas in Canada. However, interestingly the Toronto CMA appears to have an IRR of thyroid cancer that is significantly higher than those observed in the rest of the nation. We present our initial regression results in Table 1 for a sample of the larger CMAs within Canada with the Toronto CMA as the reference value.

**Table 1:** Incidence rate ratio of thyroid cancer in Canada by Census Metropolitan Area

	*IRR*	*p-value*	*CI*
*Census Metropolitan Area*			
*Toronto (omitted category)*	*1.00*		
*St. John’s*	*0.496*	*0.000*	*[0.397–0.620*
*Halifax*	*0.374*	*0.000*	*[0.313–0.446]*
*Moncton*	*0.310*	*0.000*	*[0.246–0.391]*
*St John*	*0.413*	*0.000*	*[0.335–0.508]*
*Quebec City*	*0.489*	*0.000*	*[0.438–0.544]*
*Montreal*	*0.494*	*0.000*	*[0.453–0.539]*
*Ottawa/Gatineau*	*0.403*	*0.000*	*[0.373–0.435]*
*Kingston*	*0.275*	*0.000*	*[0.222–0.342]*
*Hamilton*	*0.381*	*0.000*	*[0.347–0.418]*
*London*	*0.682*	*0.000*	*[0.623–0.747]*
*Winnipeg*	*0.493*	*0.000*	*[0.418–0.580]*
*Regina*	*0.446*	*0.000*	*[0.360–0.552]*
*Saskatoon*	*0.384*	*0.000*	*[0.310–0.475]*
*Calgary*	*0.570*	*0.000*	*[0.504–0.645]*
*Edmonton*	*0.484*	*0.000*	*[0.427–0.549]*
*Vancouver*	*0.518*	*0.000*	*[0.449–0.598]*
*Victoria*	*0.518*	*0.000*	*[0.432–0.621]*

After controlling for demographic and socio-economic factors, every one of the 150 CMAs and CAs identified had a lower incidence of thyroid cancer than the Toronto CMA. For the larger CMAs listed above, the IRR for thyroid cancer was statistically significantly lower than the Toronto CMA in all cases. In fact, for 70 % of CMAs and CAs across Canada, the IRR for thyroid cancer was less than half the IRR for thyroid cancer in the Toronto CMA.

In Table 2 we added a set of binary indicator variables for each CSD within the Toronto CMA in order to examine how incidence of thyroid cancer varies within this large area. The Toronto City core CSD is the reference category and as such all other areas were compared directly to it.

**Table 2:** Incidence rate ratio of thyroid cancer incidence by Census Subdivision in the Toronto Census Metropolitan Area

	*IRR*	*p-value*	*CI*
*Census subdivision*			
*Toronto City core*	*1.00*		
*Pickering*	*0.812*	*0.027*	*[0.675–0.976]*
*Ajax*	*0.946*	*0.555*	*[0.785–1.14]*
*Uxbridge*	*0.248*	*0.000*	*[0.127–0.486]*
*Vaughn*	*1.457*	*0.000*	*[1.297–1.637]*
*Markham*	*1.305*	*0.000*	*[1.171–1.454]*
*Richmond Hill*	*1.167*	*0.027*	*[1.018–1.339]*
*Aurora*	*0.843*	*0.204*	*[0.648–1.097]*
*Mississauga*	*0.883*	*0.003*	*[0.814–0.957]*
*Brampton*	*0.792*	*0.000*	*[0.710–0.882]*

It can be seen that there was significant variation in incidence across CSDs within the greater Toronto CMA with most CSDs exhibiting a lower incidence rate then Toronto City Core. However three CSDs had a significantly higher incidence of thyroid cancer than Toronto City: Markham (IRR 1.305, *p* = 0.000, [CI 1.171–1.454]) Vaughn (IRR 1.457, *p* = 0.000, [CI 1.297–1.637]) and Richmond Hill (IRR 1.167, *p* = 0.027, [CI 1.018–1.339]).

## Discussion

The objective of our study was to further explore the relationship between geography and incidence of thyroid cancer in Canada. Our data demonstrates that there were dramatically higher rates of thyroid cancer incidence in the Toronto CMA compared to all other CMAs in Canada, as well as when the Toronto CMA was compared to all CAs across the country. Our subset analysis also revealed that incidence varied significantly within the Toronto CMA, with a contiguous area of three regions north of Toronto (Markham, Vaughn, and Richmond Hill) showing dramatically higher incidence rates. This effect was statistically significant even after controlling for age, gender, and socioeconomic differences between populations.

Our results were similar to a recent retrospective study performed by Hall et al. [15]. The authors examined the incidence of thyroid cancer in Ontario between 1999 and 2008 between the different LHINs and compared these to rates of diagnostic test employment in these regions. They found that the rate of thyroid cancer in Ontario nearly doubled over the previously stated time interval from 10.19 to 18.89 per 100,000 population. They also found that certain LHINs within Ontario had incidence rates that were significantly higher than others. They linked these areas with rates of diagnostic imaging including ultrasound and found that the regions with the highest incidence were also those with the highest average number of ultrasounds ordered per person. They concluded that in Ontario a driving force behind the increasing rates of thyroid cancer was potential over-usage of imaging tests in certain regions.

Previously it has been shown that the observed increase in incidence of thyroid cancer is related to detection of subclinical disease and thus overemployment of screening tests could be perpetuating this cycle [17]. Hall et al. found that in Ontario the highest incidence of thyroid cancer occurs in LHIN 8; this LHIN contains the CSDs of Vaughn, Markham, and Richmond Hill, which directly corroborates our data. For example, the incidence rate of thyroid cancer in LHIN 8 was approximately 4 times as high in LHINs 10 and 11 (South East and Champlain LHINs). LHIN 8 is also the LHIN which has the highest rate of ultrasound usage in Ontario [15]. However, we do not at this time have direct evidence to support the hypothesis that there is more access to neck imaging in Toronto than in other major Canadian metropolitan areas.

While access to medical imaging may play a role in this evident disparity in thyroid cancer incidence in Toronto compared to the rest of the country, it likely does not completely explain this disparity. A number of other factors could be theorized to contribute to the effects seen including the presence of at risk groups. One factor could be ethnicity; there are certain ethnic populations in which thyroid cancer has been seen with greater frequency. An example of this is the Filipino population. Filipino women born in the Philippines had 3.2 [CI 2.7,3.8] times the rate of thyroid cancer of US-born Caucasian women [18]. The majority of immigrants to Canada, Filipino immigrants included, do settle in the Greater Toronto Area (GTA) [19]. Disparities in the distribution of certain ethnic groups predisposed to thyroid cancer may help to explain some of the marked differences in incidence seen in the Toronto CMA.

Another factor that could have been considered is exposure to ionizing radiation. Ontario is home to several large nuclear power plants, mostly concentrated around the GTA. However, no increased incidence of DTC was seen in Pickering, in which one of these nuclear plants is located which discounts this theory. Other environmental factors could also be considered.

There are several important limitations in our study. We did not posses individual-level income data and thus we used DA-level information on household income as a substitute. While household income reflects income from all sources including pensions and investment income, SES could be measured more accurately with individual level information on other determinates of SES such as education level and occupation. Income alone may be biased in favor of current SES rather than long term SES.

## Conclusions

Even after controlling for variables such as age, gender, socio-economic status, and population density, our study of the incidence of thyroid cancer in Canada from 1992 to 2007 demonstrated a dramatically higher incidence in the Toronto Census Metropolitan Area when compared to any other CMA or CA in Canada. A more detailed analysis of the Toronto CMA demonstrated that three contiguous geographical regions (Markham, Vaughn, and Richmond Hill) had significantly higher incidence rates of thyroid cancer than did the remainder of the Toronto CMA. While access to diagnostic imaging may explain part of this discrepancy in thyroid cancer incidence, other as yet unconfirmed factors may also be contributory to the increased incidence of thyroid cancer in Toronto.

## Abbreviations

InQ: Income quintiles
CMA: Census metropolitan area
CSD: Census subdivision
DTC: Differentiated thyroid cancer
SEER: Surveillance Epidemiology and End Result
LHIN: Local health integrated network
CCR: Canadian Cancer Registry
NB-RDC: University of New Brunswick Research Data Centre
CY: Densus year
DA: Dissemination area
CD: Census division
PCCF+: Postal Code Converter File
GTA: Greater Toronto area
